# Smoking cessation in pregnant women using financial incentives: a feasibility study

**DOI:** 10.1186/s12884-022-05292-9

**Published:** 2022-12-24

**Authors:** T. A. Kroder, L. L. Peters, A. L. Roggeveld, M. Holtrop, L. Harshagen, L. M. Klein, J. J. H. M. Erwich

**Affiliations:** 1 Department of Obstetrics and Gynaecology, University Medical Centre Groningen, University of Groningen, Groningen, The Netherlands; 2 Department of General Practice and Elderly Care Medicine, University Medical Centre Groningen University of Groningen, Groningen, The Netherlands; 3 Department of Midwifery Science, Amsterdam Public Health Research Institute, Amsterdam UMC (location Vumc), Amsterdam, The Netherlands

**Keywords:** Financial incentives, Feasibility study, Smoking cessation, Pregnancy, Contingent, Public acceptability, Intervention, Cotinine

## Abstract

**Background:**

The high prevalence of smoking pregnant women in Dutch areas with lower socioeconomic status and the consecutively harmful exposure to tobacco to both mother and child, depicted a high need for a novel intervention. According to other studies, the utilisation of financial incentives appeared to be a promising method for smoking cessation in pregnant women. Therefore, the aim of this study was to investigate the feasibility of implementing contingent financial incentives as smoking cessation support for pregnant women in the Netherlands.

**Methods:**

Feasibility study consisting of four developmental phases: (1) acceptability of Dutch population regarding financial-incentive-intervention by conducting an online questionnaire, (2) composing a pilot study utilising the financial-incentive-intervention in clinical practice, (3) execution of the composed pilot study and (4) evaluation of the executed pilot study utilising a mixed-methods approach.

A financial-incentive-intervention, given in a contingent financial scheme (during five consequential appointments, respectively €25/€50/€100/€150/€250), if smoking abstinence was proven by the amount of cotinine in the urine of the pregnant women measured utilising a urine dipstick test.

The public acceptability for the financial-incentive-intervention was assessed using 5-Likert scales. The number of pregnant women able to abstain from smoking during the pilot study and utilising the financial-incentive-intervention in clinical practice were used to assess the prosperity and practicality of the pilot study respectively. The pilot study was evaluated using a mixed-methods approach.

**Results:**

In total, 55.1% of the Dutch population sample (*n* = 328) found a financial incentive inappropriate for smoking cessation in pregnant women, while the healthcare professionals and pilot study participants thought the financial-incentive-intervention to be a helpful approach. Eleven vouchers were given during the pilot study, and one woman completed all test points and tested negative for cotinine at the end of the pilot study.

**Conclusion:**

Although the financial-incentive-intervention appeared to be a promising approach for smoking cessation in pregnant women, the acceptability of the Dutch population and the number of pregnant women able to abstain smoking during this pilot study was low. Despite the limited study population, this study proved the concept of this financial-incentive-intervention to be feasible for implementation in the Netherlands.

**Trial registration:**

Not applicable since this is a feasibility study prior to a trial.

**Supplementary Information:**

The online version contains supplementary material available at 10.1186/s12884-022-05292-9.

## Background

Exposure to tobacco during pregnancy is harmful to both mother and child. Smoking during pregnancy increases the risk of short- and long term complications, such as miscarriage, pre-term birth, and the development of lung diseases like asthma in the child’s later life [1–3].

In the Netherlands, several smoking cessation interventions support pregnant women to stop smoking and preventing them from relapsing after their pregnancies. In the Trimbos Guideline Smoking cessation counselling, these interventions can be provided by several healthcare professionals and are divided into psychosocial interventions (e.g. personal counselling, either face-to-face or by telephone) and pharmacological interventions (e.g. nicotine replacements) [4, 5]. Although the overall percentage of pregnant women smoking in the Netherlands has decreased in the last couple of years, the prevalence remains higher in areas with lower socioeconomic status (SES), like the three Northern provinces (Groningen, Friesland, and Drenthe) [6–8]. The province of Groningen, for example, showed varying percentages per municipality from 5.5 to 19.8% in 2019, while the overall percentage of pregnant women who smoked at any point during their pregnancy in the Netherlands was 7.4% in 2018 [9–11]. These high percentages demonstrate a high need for a novel and innovative method that is more effective among these groups. A promising method is giving financial incentives for the smoking cessation of pregnant women. Studies conducted in the United States, the United-Kingdom and France reported that this method stimulates women to quit smoking and decreases the overall percentage of women smoking during and after pregnancy [12–15].

A study conducted in the United-Kingdom showed that women who received financial incentives were more likely to quit smoking during (22.5% vs 8.6%) and after (15.0% vs 4.0%) their pregnancy compared to women who received standard intervention methods (OR 2.63; 95% CI 1.73–4.01) [13]. The women participating in this study received incentives with a maximum of 400 pounds (converted being approximately €467) per participant, and the study used carbon monoxide breath tests and cotinine saliva assays to confirm the participants’ non-smoking status. A meta-analysis demonstrated that contingent incentives also have a higher motivating effect and resulted in a larger group of participants who stopped smoking compared to non-contingent incentives (21.4% vs 5.9%, respectively) [12]. Additionally, a recent study conducted by Tappin et al. (2022) showed that, along with an increased percentage of pregnant women able to quit smoking, financial incentives are cost effective and potentially save health care costs for the society in the future [15].

Notwithstanding the positive outcomes of using financial incentives, it seems that other factors that could potentially influence the results of such an intervention should be investigated before implementing it. One factor that could negatively influence the intervention’s execution is the public acceptability in the society where the intervention will be implemented. Other studies have reported the importance of investigating public acceptability in order to implement such an intervention successfully in clinical practice [16, 17]. To our knowledge, no other study has investigated the public acceptability of the Dutch population about financial incentives for smoking cessation in pregnant women.

Concluding, this study aimed to investigate the feasibility of implementing contingent incentives as smoking cessation support in the Northern Netherlands. The project contained four developmental phases to ensure optimal implementation of the financial-incentive-intervention in clinical practice. Firstly, the Dutch population’s public acceptability for financial incentives purposed for pregnant smoking women was assessed by conducting an online questionnaire. Secondly, this study attempted to develop a financial-incentive-intervention that stimulates pregnant women to quit smoking and thirdly executed the implementation in clinical practice. Fourthly, the financial-incentive-intervention was evaluated after implementation in clinical practice, and this included both the perspectives of healthcare professionals and participants themselves.

## Methods

### Feasibility study

The theory of Bowen et al. [18] was used to assess the feasibility of this implementation study of contingent incentives for smoking cessation support [18]. This theory consists of four developmental phases; acceptability, composing, implementation and evaluation of the feasibility in clinical practice. The first phase comprised exploration of the Dutch population’s perspectives regarding the financial-incentive-intervention by conducting an online questionnaire. These results provided input for the consecutive second phase, in which a study design was composed for a pilot study, including the financial-incentive-intervention. The third phase comprised the execution of this pilot study, followed by the fourth phase, which incorporated an evaluation of the pilot study in clinical practice, utilising both healthcare professionals and pregnant women’s perspectives.

### Phase 1: acceptability

With an online questionnaire, the public acceptability of the Dutch population for the financial-incentive-intervention was assessed. The online questionnaire comprised 23 items regarding the opinion of the financial-incentive-intervention and its actual implementation. The online questionnaire was developed via Qualtrics, and the anonymous link was distributed amongst the Dutch community through 5 personal Facebook sites of students of Medical Sciences to 59 of their acquaintances or unknowns. Facebook was chosen as medium, since it is fairly easy to recruit via this way and it can be used to create a large reach in the Netherlands among different population groups. The items of the online questionnaire included demographic characteristics and statements of smoking cessation support, we did not include validated measures. The questionnaire was 3 months accessible for responding.

The questionnaire comprised of eight sociodemographic questions (e.g. gender, education, and smoking status), 10 statements concerning smoking cessation support for pregnant women (e.g. “Pregnant women may receive financial incentives if they stop smoking during their pregnancy”) and five questions concerning the design of a financial-incentive intervention (e.g. “In what form should the financial incentive be given?”). The 10 statements could be scored according to a 5-Likert scale, ranging from “strongly agree” to “strongly disagree” (see Additional file 1).

The online questionnaire results were analysed by using descriptive statistics. Statistical differences between different sociodemographic groups (gender, age, education, smoking status, and (former) pregnant women and their partners) were calculated by Student T-tests, ANOVAs, Mann-Whitney U and Kruskal-Wallis tests, where appropriate. The data were analysed utilising SPSS Statistics version 23.0 (SPSS Inc., Chicago, IL, USA).

### Phase 2: composing a pilot study

Based on a similar study by Cahill et al. (2015) on incentives for smoking cessation, the financial incentives were offered in vouchers that could be spent in a widely known baby store in multiple cities spread across the Netherlands [12]. The financial incentives would be provided to the recruited pregnant women during five scheduled appointments if the measured amount of cotinine proved smoking abstinence in the urine of the pregnant women by means of a urine dipstick test (Drug test Drug-Detect Nicotine (Cotinine), 200 nanogram/mL) [19]. Studies described cotinine urine dipstick tests as a valid means of detecting smoking cessation in participants [20, 21]. Furthermore, these studies showed that cotinine in the urine could still be detected for two to 4 days after smoking, enabling more reliable detection of the smoking status of the included pregnant women in our study.

**Fig. 1:**
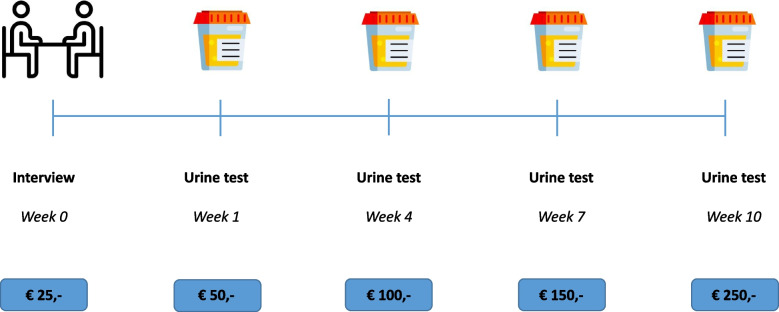
Study design for the pilot study. Illustrating the time points of the interview and cotinine urine tests plus the amount of money the participants could receive at each time point. The green cotinine urine tests represent negative tests indicating no exposure to smoking. The Figure illustrates the amount of money the vouchers would represent if a participant would be present at the interview and consecutively would have four negative cotinine urine tests indicating she had stopped smoking. In case a participant would have a positive cotinine urine test in week 7, this would result in no voucher at that appointment and consequently a final voucher of €150 euros in week 10 if the cotinine urine test would then again be negative

The pilot study’s financial scheme was based on both the results of Phase 1 and on published literature [12, 14]. The results of Phase 1 were used to support the amount and form of the voucher, while the contingent scheme was based on efficacy as proven in recent literature. A €25 voucher could be obtained during the first study appointment, which included a cotinine urine test plus a semi-structured interview to gain more information about the participants’ sociodemographic characteristics and factors that could influence smoking cessation, such as home situation and social support. The four consecutive appointments primarily encompassed cotinine urine tests, for which €50/€100/€150/€250 vouchers could be obtained respectively, bringing the total amount to be received in vouchers up to €575,-. The vouchers were given according to a contingent financial scheme, meaning the amount of money increased each time a pregnant woman tested negative for the cotinine urine test, as shown in Fig. 1. The contingent financial scheme was meant to stimulate women for a longer period of time, as this would result in a higher reward. The voucher could not be obtained if the pregnant woman tested positive, implicating the total amount of money could not be reached anymore by this woman. The study appointments were scheduled with two researchers at the University Medical Center Groningen (UMCG), and were preferably combined with their usual care appointments. The appointments were aimed to be scheduled every 3 weeks, consecutive leading to the pilot study lasting approximately 10 to 14 weeks per participant.

### Phase 3: execution pilot study

The pilot study implemented the financial-incentive-intervention in clinical practice. The smoking pregnant women were recruited in the city of Groningen at three locations, a universal hospital (University Medical Centre Groningen; UMCG), a general hospital (Martini Hospital Groningen; MZH), and a primary-care midwifery practice (Verloskundige Stadspraktijk Groningen; VSP). The women were recruited in the period March–May 2019.

Healthcare professionals (midwives, medical nurses, *n* = 9), who were employed at the abovementioned locations, identified eligible smoking pregnant women at their first appointment and informed them about the pilot study. In case of the women’s eligibility and interest, the researchers contacted them to give further information and recruit them afterwards. Informed consent was given by each participant before starting the study and afterwards appointments were scheduled for the study and registered as “Visit 1 to 5”.

Women were included if they were older than 17 years of age, gestational age < 30 weeks, self-reported smoking at the time of the first appointment, and were motivated to quit smoking. Pregnant women utilising nicotine replacements or e-cigarettes were excluded, as previous studies stated that these forms of nicotine could be harmful during pregnancy [22–24]. A recent study by Hajek et al. (2022) showed that the utilisation of nicotine replacements and e-cigarettes leads to health risks for both mother and child and maintains the nicotine dependence [24]. These nicotine replacement therapies could also intervene with the cotinine urine tests and would therefore be a confounding factor when assessing smoking status of the pregnant women.

The study protocol was approved by the Medical Ethics Research Committee of the UMCG (nr 201,800,826). The study was conducted in accordance with Good Clinical Practice guidelines and regulations.

### Phase 4: evaluation of pilot study

The pilot study was evaluated using a mixed-methods approach. Firstly, five healthcare professionals, who were involved during the execution of the study, were interviewed about their experiences with the financial-incentive-intervention pilot study. During these semi-structured interviews their opinion about the financial-incentive-intervention in daily practice was asked. Next, the experiences of all involved healthcare professionals, affiliated at the three hospitals, were collected during these interviews assessing the feasibility of the pilot study and the possible implementation aside the usual guidelines for smoking cessation for pregnant women.

Next, a standardised questionnaire was utilised to obtain data about the included pregnant women’s pilot study experiences. During the participant’s last study appointment, the pregnant women were invited for their last cotinine urine test and asked to complete a standardised questionnaire. This questionnaire contained items about experiences during the pilot study’s inclusion and consecutive execution, their opinion about financial incentives for smoking cessation for pregnant women, and their suggestions for future studies.

Moreover, all included women were asked to participate in a semi-structured interview at the first study appointment. During the interviews women’s individual experiences with smoking cessation interventions were discussed even as their smoking behaviour, pregnancy and social status. Also, the support and perceptions of their partners regarding smoking cessation interventions were discussed, if applicable. The interviews were digitally recorded and transcribed verbatim and analysed utilising ATLAS.ti (ATLAS.ti Scientific Software Development GmbH. ATLAS.ti for Windows, version 5.2.18) [25].

Furthermore, the prosperity of implementing the financial-incentive-intervention in clinical practice was assessed at the end of the pilot study by affirming the number of pregnant women able to abstain from smoking during the pilot study. Lastly, an evaluation of the pilot study’s practicality was performed by looking in retrospect at the number of women using the financial-incentive-intervention in clinical practice.

## Results

### Phase 1: acceptability

**Fig. 2:**
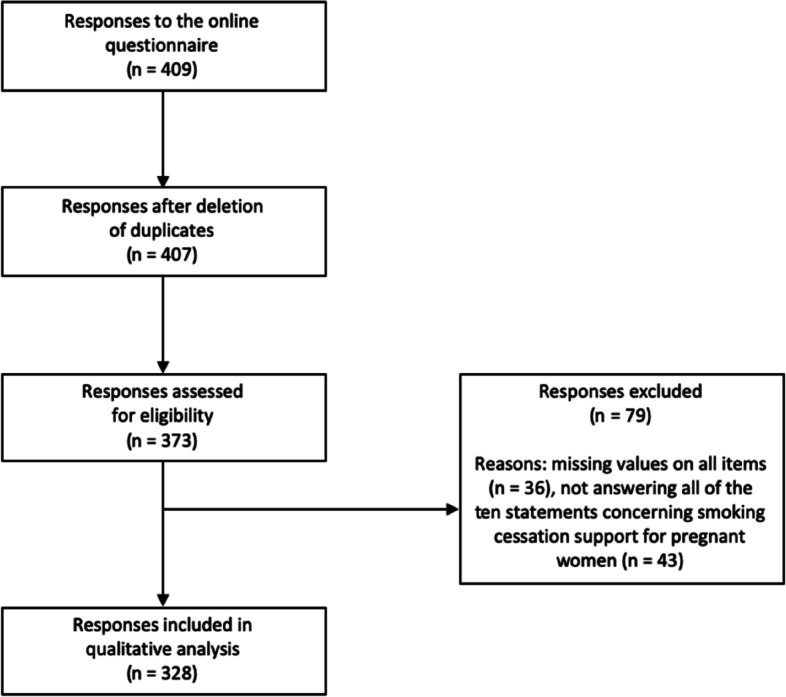
Flowchart inclusion of responses to the online questionnaire

In total, 409 individuals responded to the online questionnaire. Figure 2 shows a total of 328 responses were included in the analyses to assess the Dutch population’s acceptability of financial incentives for smoking cessation in pregnant women. In total 81 responses were excluded from the analysis due to duplications (*n* = 2), missing values on all items (*n* = 36) or not answering all of the 10 statements concerning smoking cessation support for pregnant women (*n* = 43).

Table 1 shows the respondent’s characteristics. The majority of the respondents were female (*n* = 266, 81.1%) and between 20 and 29 years (*n* = 133, 40.5%). Most of the respondents had high education (*n* = 213, 64.9%) and had never been pregnant (*n* = 194, 59.1%). Furthermore, approximately a quarter of the respondents or their partners were smoking (*n* = 86, 26.2%) and another quarter had smoked in the past (*n* = 80, 24.4%). Almost half of the respondents who were smoking before their pregnancy were able to quit smoking, of whom 39.1% (*n* = 34) only stopped during the pregnancy and 13.8% (*n* = 12) permanently stopped smoking. The other respondents did either smoke some part of their pregnancy (*n* = 23, 26.4%) or did not stop at all (*n* = 18, 20.7%).

**Table 1:** Sociodemographic characteristics of respondent’s participating in online questionnaire (Phase 1) (*n =* 328)

RESPONDENTS CHARACTERISTICS	Total population ***N =*** 328, 100% N (%)
**Gender**	
Male	62 (18.9)
Female	266 (81.1)
**Age (years)**	
< 20	22 (6.7)
20–29	133 (40.5)
30–39	52 (15.9)
40–49	66 (20.1)
50–59	36 (11.0)
≥ 60	19 (5.8)
**Education**^**1**^	
Low	13 (4.0)
Medium	102 (31.1)
High	213 (64.9)
**Pregnancy status**	
I/my partner is pregnant	3 (0.9)
I/my partner has been pregnant	131 (39.9)
I/my partner have never been pregnant	194 (59.1)
**Smoking status**	
I smoke	40 (12.2)
My partner smokes	24 (7.3)
We both smoke	22 (6.7)
I have smoked	36 (11.0)
My partner smoked	18 (5.5)
We both smoked	26 (7.9)
No, I do not smoke	65 (19.8)
No, we both do not smoke	97 (29.6)
**Stopped smoking during pregnancy**	***N =*** **87**
Stopped during pregnancy	34 (39.1)
Permanently stopped after pregnancy	12 (13.8)
Did not completely stop during pregnancy	23 (26.4)
Did not stop at all	18 (20.7)

^1^ Education is categorized the following, low education (i.e. primary education, lower vocational education, intermediate general secondary education or not completed primary school), medium education (i.e. higher general secondary education or intermediate vocational education) and high education (i.e. university of higher vocational education)

The sequent part of the questionnaire comprised 10 statements concerning smoking cessation support for pregnant women. The results showed large disunity among the respondents. Respondents indicated that they consider financial incentives a controversial intervention and are both positive and negative towards various aspects of the implementation of the financial-incentive-intervention. The importance of smoking cessation for pregnant women was affirmed by the vast majority of the respondents (*n =* 298, 90.9%). Furthermore, the majority of the respondents considered it feasible for pregnant women to quit smoking, both during and after the pregnancy (respectively *n =* 268, 82.0%, and *n =* 238, 72.8%). In total, 170 (51.8%) of the respondents indicated that they totally disagreed to disagreed with the statement for rewarding pregnant women with financial incentives if they quit smoking during their pregnancy. Additionally, half of the respondents (totally) disagreed with the possible implementation of the financial-incentive-intervention (*n =* 178, 54.3%), and a quarter (*n =* 80, 24.4%) of the respondents (totally) agreed with this statement.

The last five questions of the questionnaire concerned the design of the financial-incentive-intervention. The results showed that 55.1% of the respondents (*n =* 179) thought it was inappropriate to give a financial incentive for smoking cessation, compared to 31.7% of the respondents (*n =* 103) who did think it was an appropriate intervention and 13.2% (*n =* 43) of the respondents who were not sure whether it was appropriate or inappropriate. Approximately one third (*n =* 103, 31.4%) of the respondents thought it was feasible for the women to stop smoking using the financial-incentive-intervention, while one third (*n =* 108, 33.0%) thought it was not feasible, and one third (*n =* 117, 35.6%) did not know whether it was feasible. Furthermore, the respondents were asked who should help pregnant women stop smoking. The most chosen option was an addiction medicine physician (*n =* 114, 37.9%), followed by a general practitioner (*n =* 54, 17.9%), a midwife (*n =* 45, 15.0%), a general practice-based nursing specialist (*n =* 42, 14.0%), a gynaecologist (*n =* 13, 4.3%), a combination of healthcare professionals and family or partner, (*n =* 12, 4.0%) and the pregnant woman herself (*n =* 9, 3.0%).

In case the financial-incentive-intervention would be implemented, most respondents felt the amount of money should be between €0–€100 (*n =* 32, 31.1%), between €100–€200 (*n =* 33, 32.0%) or between €200–300 (*n =* 16, 15.3%). Furthermore, it was believed by a large group of respondents that the financial incentives could best be given in the form of vouchers (*n =* 120, 38.6%). Around half of the respondents (*n =* 158, 49.8%) believed it would then be best to give the complete reimbursement at the end of the pilot study, while 37.5% (*n =* 119) believed the reimbursement should be incremental amounts during the pilot study and 12.6% (*n =* 40) believed the reimbursement should be distributed equally during the pilot study.

### Phase 2: composing a pilot study

Based on the composed study design, the pilot study lasted 10 to 14 weeks per participant. We aimed to schedule the visits of the pregnant women every 3 weeks. However, the majority (*n =* 6, 85.7%) cancelled their appointment frequently, which resulted in a different visiting schedule than the proposed study design.

### Phase 3: execution pilot study

In total, nine pregnant women were included in the pilot study at three different locations. Five women were recruited at the university hospital (UMCG), two women at the general hospital (MZH), and two women at the primary midwifery practice (VSP). Table 2 illustrates the number of conducted interviews, dropouts, and negative and positive cotinine urine tests during the pilot study. Two women dropped out before conducting the interview at the first appointment, and six women dropped out before the end of the pilot study. One woman completed all test points and tested negative at the end of the pilot study, despite having a positive test at test point 3.

**Table 2:** Numbers of included pregnant women, conducted interviews, dropouts, negative cotinine urine tests and positive cotinine urine tests during the pilot study

	INCLUSION	VISIT 1	VISIT 2	VISIT 3	VISIT 4	VISIT 5	TOTAL
		Interview	Urine Test	Urine Test	Urine Test	Urine Test	
**Participating women (N)**	9	7	6	3	2	1	NA^1^
**Women dropped out (N)**	0	2	1	3	1	1	8
**Negative Urine Test (N)**	NA	NA	2	1	0	1	4
**Positive Urine Test (N)**	NA	NA	4	2	2	0	8

^1^ Not applicable

During the interviews at the start of the pilot study, it was found that all of the pregnant women were unemployed (*n =* 7, 100.0%), all had attempted to stop smoking in the past (*n =* 7, 100.0%), and most of them had health issues (*n =* 6, 85.7%)(Table 3). The majority of the women started smoking at a young age, contributing to an average of 15 years of smoking, and the majority (*n =* 5, 71.4%) also smoked inside their houses. Furthermore, the majority of women endured problems in their private situations, such as financial issues (*n =* 5; 71.4%) or relational issues with partner or family (*n =* 5; 71.4%). There were no distinctive differences between the case of the women who completed the pilot study and the women who dropped out.

**Table 3:** Characteristics of women at the start of the pilot study who participated in semi structured interviews (*n =* 7)

CHARACTERISTICS PREGNANT WOMEN	N (%)
**Age (years)**	
20–25	3 (42.9)
26–30	1 (14.3)
31–35	2 (28.6)
36–40	1 (14.3)
**Pregnancy**	
First	2 (28.6)
Second	3 (42.9)
Third	2 (28.6)
**Gestation (weeks)**	
8 + 0–10 + 6	2 (28.6)
11 + 0–13 + 6	2 (28.6)
14 + 0–16 + 6	1 (14.3)
17 + 0–19 + 6	0 (0.0)
20 + 0–22 + 6	1 (14.3)
23 + 0–25 + 6	1 (14.3)
**Employment**	
Yes	0 (0.0)
No	7 (100.0)
**Years of smoking**	
< 5 years	0 (0.0)
5–10 years	1 (14.3)
10–15 years	4 (57.1)
> 15 years	2 (28.6)
**Previous smoking cessation attempt**	
Yes	7 (100.0)
No	0 (0.0)
**Private problems**	
Relational issues (with partner and/or family)	5 (71.4)
Financial issues	5 (71.4)
**Health issues**^**1**^	
Yes	6 (85.7)
No	1 (14.3)

^1^ Health issues include Crohn’s disease, HELLP syndrome, preeclampsia, hereditary disorder, gestational diabetes, and peri-gestational haemorrhage

When asked for the motivation to participate in the pilot study, the majority was motivated to participate due to the unborn child’s health. Some women also mentioned that the pilot study acted as extra motivation. All of the women had attempted smoking cessation, of which only two women (*n =* 2, 28.5%) were once successful in the past, and all repeatedly affirmed that it was difficult for them to stop smoking. Women saw addiction (*n =* 4, 57.1%), stress (*n =* 6, 85.7%) and routine (*n =* 5, 71.4%) as the main obstacles to successfully quit smoking. To illustrate this, someone quoted, *“(…) when I faced difficult times during my life, I started smoking again (…)”*(Table 4)*.* Other aspects that influenced their smoking cessation attempts were family or friends smoking in close proximity.

**Table 4:** Code tree of the conducted interviews with the pregnant women (*n =* 7) including the themes, categories, subcategories, codes, quotes and anonymised participant IDs

CODE TREE - THEMES (CATEGORY)	SUBCATEGORY	CODE	QUOTE	PARTICIPANT ID^**1**^
**Smoking**				
Positive influence on smoking cessation	Activity	Distraction	*“Usually, I get up and then I know if I, when I get up and I have breakfast and do my things first, then um, I don’t have to smoke anymore”*	U08
		Taking distance	*“... I’ve also decided not to go to friends for a while, this week anyway, so that I won’t be tempted either.”*	U08
		Eating	*“… well in the first week you just have to try to distract yourself that way ...”*	U11
		Focus	*“... when I have the ultrasound, that you can hear the heartbeat that I will become more aware of oh yes I really have something in my belly …”*	M02
		Hobby’s	*“… hobby is photography, but uh, that’s also paused at the moment. But I plan to do that again.”*	U05
		Household	*“Cleaning up, tidying up.”*	U04
		Avoidance	*“Yes, especially with my boyfriend, when I, um, know that there is really nothing in the house. That there is nothing to look for ...”*	U11
	Aid with smoking cessation	Social	*“Yes indeed, and my best friend also has 2 children, so you spend a lot of time together”*	M02
		Sport	*“… pregnancy yoga first and I want to continue that later, …, you will fully relax yourself ...”*	U05
		Approach	*“... without there being, um, a pointing finger ...”*	S12
		External support	*“… because you have to stop (…) not only my environment but eeh, also people in the hospital ...”*	U05
		Request for help	*“And now I wonder, maybe it would be like this (…) if I had perhaps had, then it might have been that I would have stopped longer”*	U04
		Internal support	*“... my friend gets angry if I smoke. But the rest of the environment is not so judgmental and angry.”*	U04
		Nicotine replacements	*“... I would like to use nicotine patches. But then if I do urine test you still have that, it still has, uh, nicotine in it.”*	U04
		Self	*“I’m really like eeh, yes I want it myself and I really support it so ehm, I can do that ...”*	U05
Negative influence on smoking cessation	Counteractions	Barrier	*“... after that there was no sympathy for the fact that I did smoke ... he reacted so angry that we got into a fight.”*	U04
		Perseverance	*“... not motivated enough to ...”*	U04
		To deny	*“And then you no longer assume things that can go wrong.”*	U08
		Rushed	*“... I’m already doing really well and then I’m going to seven milligrams, well and then I’m done with it and I overestimated myself in that.”*	U04
		Temptation	*“... then I was with friends again and then, yes, give me a cigarette too, yes then you start again.”*	U11
	Reasons to smoke	Reward	*“... because I no longer smoke weed, I still smoke, because it is so good that I did stop smoking weed. That’s my reward.”*	U04
		Relaxation	*“... now let’s relax through ...”*	U08
		Smoking thoughts	*“And then it was really every day that I still thought, I have to, I have to, I have to, I have to.”*	U04
		Routine	*“Yes, really dinner … I used to have, did I have in the morning that I thought now I’m going to have a cup of coffee and a cigarette ...”*	U05
		Peace	*“When I smoke a cigarette, I calm down too.”*	U05
		Stress	*“Then I ended up in a stressful situation again, so yes. Then you soon reach for a cigarette again.”*	U05
		Addiction	*“... because I have smoked now and then in between, you still have the taste for a bit ...”*	M02
		Boredom	*“... when you are home alone … I found it very difficult not to smoke because it was so quiet.”*	U08
		Fancy	*“... every now and then you just feel like a cigarette.”*	U05
Smoking behaviour	Location	Inside	*“… usually in the kitchen with a window open, but not in the room.”*	U08
		Outside	*“Not even on the balcony, … just go outside.”*	U05
	Tobacco products	E-cigarette	*“... electric. I did that for a while.”*	U04
		Cigarette	*“I actually smoke cigarettes.”*	U08
		Weed	*“... before my pregnancy, um, I smoked weed too ….”*	U04
Smoking cessation	Attempt to quit	Fed up	“*It is, yes, just a shame …”*	S01
		Easy	*“... it’s okay (...) I actually stopped quite easily.”*	U04
		Failed attempt	*“... I did smoke a few times again, when I faced difficult times during my life, I started smoking again”*	U05
		Hard	*“... it is very difficult for me to quit smoking.”*	U08
		Period	*“... I quit on my own for 3 months.”*	U05
		Regret	*“Because then I have those side effects and now, I also like well, I should have done this much sooner actually ...”*	U04
		Date of stopping	*“... I have something like (...) I’m going to start quitting today ...”*	U05
		Successful attempt	*“I pretty much stopped together with him, actually.”*	M02
		Proud	*“... I am kind of proud but it doesn’t feel um, I am not very proud. Because I (...) now also like, well, I should have done this much earlier actually ...”*	U04
	Consequences	Used to	*“... now that was really after 3 days that I really didn’t have that anymore.”*	U04
		Withdrawal symptoms	*“Uh, yeah the first few days it was just that I was um, really cranky.”*	U04
		Symptomatic aid	*“Yes, I still got a prescription from the midwife, um, yes, the midwife gave me, um, something.”*	U04
**Pregnancy**				
Pregnancy		Uncomplicated pregnancy	*“Yes, I am happy to have a new pregnancy. (…) But if I hear more and more things are going well, then, um, I am more relaxed myself.”*	U08
		Preparations	*“... well at home I’m still busy with the nursery of course and buying clothes and things like that, so I’m preparing well, yes.”*	U05
		Pregnancy ailments	*“If something makes you nauseous all at once and then all at once, you’ve just eaten, well everything will come out again.”*	U08
**Social**				
Economic situation		Financial	*“... because I have a benefit, after about two or 3 weeks (...) my money is normal, so low that I cannot, um, spend 20 euros on it because I still have 50 euros for groceries ...”*	U04
		Preliminary education	*“... lower vocational education, but I have been working since I was 18...”*	U05
		Employment	*“I just got back to work,so I’m going to work for the first time today.”*	U08
		Unemployed	*“... and now I stopped working again ...”*	U08
Person		Health	*“Yes and eeh, then I got eeh, Crohn’s disease …, so I have to gradually rebuild everything a bit.”*	U05
		Deceased child	*“... I also had a miscarriage before at 11 weeks ...”*	M02
		Personal	*“And, well, I found those telephone contacts, um, that I found it really difficult to be open to others”*	U04
**Research**				
Test		Negative test	*“... it is indeed negative ...”*	U04
		Positive test	*“... we had expected it a bit, unfortunately, …, but there was no second line to be seen and that actually means that there is still cotinine in the urine”*	U08
Reason to stop smoking		Purpose	*“... I just know there is, a creature living inside me, a daughter, … that stands up, really number one for me, so that’s what I’m doing it for.”*	U05
		Child	*“... then you also realize it more, there is something in my belly. There’s a creature in my belly.”*	U05
Other		Interest	*“I think it is very important, after all I have been through ...”*	U08
		Aim	*“I was already looking at second-hand but now I’m like oh, maybe I can get a new one.”*	U04
		Feedback	*“... that also feels good, I have also been in conversations that one is only talking and the other is just sitting around, so this is, I just like this.”*	U05

^*1*^ *ID = Identity Documentation, generated by the first letter of the hospital recruiting the pregnant women and the number representing the month in which they were born*

### Phase 4: evaluation of pilot study

All healthcare professionals (midwives, medical nurses, *n =* 5) were enthusiastic about the financial-incentive-intervention pilot study, both prior to as after conducting the study. Some professionals were hesitant at first, as they thought the incentives were unfair to the non-smoking pregnant women. Despite this, they all acknowledged the importance of pregnant women to stop smoking and were pleased to convey this novel approach for smoking cessation in pregnant women. Aside from the positive feedback assembled during these contacts, a few suggestions were offered for possible future implication. The healthcare professionals in the hospitals commented that they think it would be more successful to include more pregnant women from midwifery practices instead of hospitals’ inclusion. They argued that many pregnant women in the hospital are more complex due to their medical situation as well as their social situation, possibly negatively influencing the pilot study. Additionally, the midwives stated that they have a large number of smoking pregnant women in their practice. The low inclusion number was, according to them, caused by a short period of inclusion and colleagues forgetting to mention the pilot study and not due to pregnant women not willing to participate in the pilot study. Additionally, all healthcare professionals mentioned that the pilot study’s execution was not troublesome, and they had no further suggestions about the practicality of execution in retrospect.

Furthermore, pregnant women were also asked about their experiences with the pilot study, about financial incentives for smoking cessation for pregnant women in general and suggestions for future implications. Overall, the pregnant women assented that they were well informed about the study before they started. The appointments were experienced as pleasant, although some mentioned being ashamed about their smoking habits and found it hard to talk about it with healthcare professionals. All pregnant women thought of the financial-incentive-intervention as a helpful approach for pregnant women to stop smoking. Women also shared the perceptions of their partners about the financial incentives for smoking cessation and most of them (*n =* 5) were positive about the intervention, since they acknowledged the importance of stopping smoking for their unborn child and wanted their partner to be able to abstain smoking during the pregnancy. It must be acknowledged that not all partners felt the same way about this and believed that the women should be able to stop without any help, as mentioned either by themselves when present or reported by the pregnant women about their partners. Along with the study, the researchers noticed that some pregnant women (*n =* 4; 57.1%) desired additional support in the form of professional help. Therefore, they were provided with information about possible options such as support by telephone or therapy by another healthcare professional.

Moreover, the qualitative data from the conducted interviews with the nine pregnant women found that the main factors essential for the successfulness of smoking cessation were the absence of stress, stable home situation and support from the environment, as shown in Table 4.

Lastly, the prosperity and practicality of the pilot study were assessed. At the end of the pilot study, one out of nine pregnant women was able to abstain from smoking utilizing the financial-incentive-intervention. In total, 11 vouchers (7x €25,-; 2x €50,-; 1x €100; 1x €150,-) were given during the pilot study to different pregnant women, representing the financial-incentive-intervention practicality in clinical practice. In total 7 women received at least one incentive during the pilot study (77.8%).

## Discussion

This study was a feasibility study of implementing financial incentives to encourage pregnant women in the Northern Netherlands to quit smoking. During this study, it was found that it was possible to implement the pilot study at different healthcare institutions, and it appeared that participants, healthcare professionals and affiliated institutions wanted to collaborate. In the pilot study, factors negatively influencing smoking cessation were stress, home situation, and lack of support from their surroundings. In total, 55.1% of the Dutch population sample, who completed the online survey, indicated that a financial incentive was inappropriate for smoking cessation in pregnant women. Most respondents believe it is possible for pregnant women to quit and stay quit from smoking. It is interesting to see that the opinion of the Dutch population in this study shows some discrepancies compared to the literature. National data shows that 52.0% of all women who smoke before their pregnancy are able to quit smoking during the first trimester and stay quit during the entire pregnancy [9]. After giving birth 43.0% starts smoking again, of which 20.0% within 4 weeks after the delivery. However, it must be mentioned that this population sample is not representative for the Dutch population, since the majority of the respondents were women (*n =* 266, 81.1%) and due to the sampling method, which caused biased inclusions of acquaintances of Medical Sciences students. This opinion is nevertheless similar to other published research in which the utilization of financial incentives in healthcare was seen as unfair compared to standard interventions and depended on the extent to which the target group was seen as responsible for their smoking behaviour [12, 19]. In addition, it was also seen that respondents with a younger age, women, smokers who tried to quit, and respondents with a higher education answered significantly more positively to the 10 statements, of which only age was found to be significant in our study [12].

However, other studies with pregnant women who smoke and the use of incentives as motivation to quit have indicated that this is a successful intervention [10, 11, 20–24]. One study found that smoking pregnant women mainly had smoking cessation problems due to environmental barriers, mainly from family smoking status, which was also found in our study [26].

This study had several limitations. Firstly, the limited respondent group did not correctly represent the population in our study’s several phases. The pilot study participants were mainly unemployed pregnant women with low SES, which could indicate that this population benefits the most from the financial-incentive-intervention. Although low SES was represented during the pilot study, this population was not appropriately represented in the online questionnaire, which was mainly answered by respondents with a high SES [26].

The limited group size also applies to the pilot study participants, which was estimated higher prior to the study. A short inclusion period and inclusion of pregnant women from the two hospitals were the main recruitment barriers that were faced during this study. The recruitment from the primary-care midwifery practice started late in the process of inclusion and resulted a low inclusion of healthy pregnancy women. The limitations may have led to inclusion bias, in which only pregnant women of lower socioeconomic class or with a medical illness wanted to participate. Inclusion bias might also have occurred in the questionnaire, in which the most outspoken respondents may have taken part. Furthermore, due to the small sample size, no statement could yet be made about the prosperity of implementing the financial-incentive-intervention in clinical practice. Moreover, it is hard to conclude about the prosperousness of the sample size in future studies, since the total number of approached and eligible women were not collected during this study. It is therefore not known how many eligible participants have been missed.

In addition, it should be considered to provide education for the healthcare workers or clinicians to gain further acceptance of contingency intervention programs in the future as this could contribute to higher inclusion rates in future studies. It was noted during this pilot that healthcare workers who were involved in this pilot study were hesitant at the start, as they thought the incentives were unfair to the non-smoking pregnant women, which might have caused them to include less women for our pilot study.

Secondly, this study has a high dropout rate as only one participant completed the study. There were no distinctive differences established that could explain the successfulness of this participant compared to the dropouts. The majority of the participants who dropped out (*n =* 8, 62.5%) did not show up to or cancelled the scheduled appointments. During the pilot study, it appeared that as a result of the cancellations and the consecutively rescheduling, the duration between the different appointments was more than the desired 3 weeks, occasionally resulting in noncompliance with the study design. It often turned out to be rather challenging to contact the concerned participants, resulting in no follow-up appointments. A review by Dantas et al. on contributing factors to missing appointments found that the use of tobacco and young patients with low socioeconomic status living far away from the clinic are important contributing factors [27]. According to the authors, it is advisable to plan as many appointments as possible in advance for participants where the risk is high. Although this has also been tried as much as possible in this study, it may be useful to pay more attention to this in a follow-up study. In addition, several participants mentioned they stopped because missing the last reimbursement due to a positive cotinine urine test took away the motivation, and the study was experienced as stressful. The stress of the study was in addition to the stress that many participants experienced because of their private situation and the medical complications during their pregnancy. This exposure to stress raises questions about whether it might be better to give a fixed amount for each appointment instead of an incremental amount, whether the interval between amounts should be reduced, or whether more appointments should be scheduled. Follow-up studies should examine the possibility of providing a show-up fee in addition to the current contingent incentives in order to decrease the dropout. A study by Berlin et al. utilized a standard voucher of €20,- at the end of each visit as a show-up fee irrespectively of smoking status [14]. This might motivate pregnant women more to continue participating in the study when they are not able to quit smoking or stay quit. This study provides a suitable and effective study design comparable to our study design, which can be used by future studies to implement the financial-incentive-intervention in the Netherlands. Additionally, it should be considered to utilize vouchers that can be redeemed in a large number of different stores instead of a baby store voucher. Changing the voucher could increase the value of the voucher for all the included pregnant women utilizing financial incentives and thereby perhaps increase the sample size and decrease the dropout.

Moreover, it should be considered to offer the pregnant women standardised additional support in the form of professional help, such as telephone support or specialized addiction care support, to be incorporated in the study design of the financial-incentive-intervention [13–15, 28]. Literature states that incorporating these interventions, mainly focused on behavioural counselling, improve the overall outcomes even more. In the Netherlands, these interventions are the usual support for smoking cessation for pregnant women. If the financial-incentive-intervention would be imbedded in the usual care, it would possibly also lead to less dropout.

## Conclusions

In conclusion, this study offered promising opportunities for smoking cessation in pregnant women to reduce the consecutive harmful impact on the health of both the pregnant women and their unborn child. Acceptability is a critical point for further continuation of this study, both from the population, where there was an adverse reaction to rewarding the participants, as from the pregnant women, where little was either achieved with smoking cessation during this study. Notwithstanding, it can be said that practicality and implementation have been met on various points of feasibility. In defiance of the limited study population this study demonstrated the proof of concept of the financial-incentive-intervention to be feasible for implementation. A follow-up study needs to be performed to confirm this pilot’s study feasibility and prove the effectiveness of the financial-incentive-intervention in a larger study population, which could be achieved by tackling the aforementioned limitations. It would then be important to recruit more pregnant women at primary-care midwifery practises, so pregnant women with and without medical concerns are equally represented in the study population. Furthermore, standardised additional professional help should be incorporated in the study design to provide additional support in smoking cessation, reduce the exposure to stress, and possibly prevent the high number of dropouts. Dropouts should further be limited by scheduling more appointments in advance to avoid high number of no-shows. If future studies would then prove the effectiveness of the intervention to reduce the number of smoking accordingly reduce the high prevalence of pregnant women smoking in Dutch areas with lower socioeconomic status and diminish the consecutively harmful exposure to tobacco to both mother and child.

## Supplementary Information

**Additional file 1.**

## Data Availability

All data generated or analysed during this study are available from the corresponding author on reasonable request.

## Abbreviations

SES: Socioeconomic status
UMCG: University Medical Centre Groningen
MZH: Martini Hospital Groningen
VSP: Verloskundige Stadspraktijk Groningen
