# Supplementary material for: Smoking cessation in pregnant women using financial incentives: a feasibility study
Source: BMC Pregnancy Childbirth. 2022 Dec 24;22:963. doi: 10.1186/s12884-022-05292-9 (PMC9789602; doi:10.1186/s12884-022-05292-9)
Supplement: Supplementary file 1 — Additional file 1. [file 12884_2022_5292_MOESM1_ESM.docx]

# **SUPPLEMENTARY TABLE**

Table S1. Ten statements concerning smoking cessation support for pregnant women of the online questionnaire and five questions concerning the design of the financial-incentive intervention (Phase 1).

|  |
| --- |
| **STATEMENTS^1^** |
| 1. “It is important that pregnant women stop smoking during their pregnancy.” 2. “It is important that pregnant women keep abstinent from smoking both during and after their pregnancy.” 3. “It is feasible for pregnant women to stop smoking during their pregnancy.” 4. “It is feasible for pregnant women to keep abstinent from smoking both during and after their pregnancy.” 5. “Pregnant women may receive financial incentives if they stop smoking during their pregnancy.” 6. “Pregnant women may receive financial incentives if they keep abstinent from smoking both during and after their pregnancy.” 7. “The partner should receive a financial incentive if he/she stops smoking in the surrounding of the pregnant women and/or infant.” 8. “Pregnant women may receive an extra financial incentive if they stop smoking together with their partner.” 9. “The financial incentives that pregnant women would receive should be income-related.” 10. “An intervention consisting of financial incentives given to pregnant women who stopped smoking should be implemented in the Netherlands.” |
| **STUDY DESIGN QUESTIONS** |
| 1. “Who should help a pregnant women to quit smoking?”    1. General practitioner;    2. Gynaecologist;    3. Addiction medicine physician;    4. Midwife;    5. General practice-based nursing specialist;    6. Other, namely *[…]^2^*. 2. “What would you consider an appropriate reimbursement for women who quit smoking during and after their pregnancy?”    1. €0 - €100;    2. €100 - €200;    3. €200 - €300;    4. €300 - €400;    5. €400 - €500;    6. More than €500;    7. I don’t think a reimbursement is appropriate;    8. I don’t know. 3. “If reimbursements would be given, in what form should they be offered?”    1. Cash;    2. Transfer to bank account;    3. Gift cards;    4. Other, namely *[…]^2^*. 4. “If reimbursements would be given, what would you think is an appropriate way to give the reimbursement?”    1. The complete reimbursement at the end of the pilot study;    2. Divide the reimbursement equally during the pilot study;    3. Provide the reimbursement in increasing amounts during the pilot study. 5. “Do you think it is feasible for women to quit smoking with the use of financial incentives?”    1. Yes, because *[…]^2^*;    2. No, because *[…]^2^*;    3. I don’t know, because *[…]^2^*. |

^1^ The ten statements could be scored according to a 5-Likert scale, ranging from “strongly agree” to “strongly disagree”.

^2^  These answer options could be self-completed by the respondent.
